# Pharmacokinetic Study of 14-(3-Methylbenzyl)matrine and 14-(4-Methylbenzyl)matrine in Rat Plasma Using Liquid Chromatography-Tandem Mass Spectrometry

**DOI:** 10.1371/journal.pone.0116010

**Published:** 2015-02-25

**Authors:** Minjie Jiang, Lisheng Wang, Shulin Huang, Liba Xu, Chao Hu, Weizhe Jiang

**Affiliations:** 1 School of Chemistry and Chemical Engineering, Guangxi University, 100 Daxue Road, Nanning, 530004, China; 2 School of Pharmaceutical, Guangxi Medical University, Nanning 530021, China; University of Edinburgh, UNITED KINGDOM

## Abstract

A rapid, sensitive and selective high-performance liquid chromatography-tandem mass spectrometric method (HPLC-MS) was developed and validated to determine the 14-(3-methylbenzyl)matrine (3MBM) and 14-(4-methylbenzyl)matrine (4MBM) levels in rat plasma in the present study. The analytes were separated using a C18 column (1.9 μm, 2.1 mm × 100 mm) equipped with a Security Guard C18 column (5 μm, 2.1 mm × 10 mm), followed by detection via triple-quadrupole mass spectrometry using an electrospray ionization (ESI) source. Sample pretreatment involved one-step protein precipitation with isopropanol:ethyl acetate (v/v, 25:75), and pseudoephedrine hydrochloride was used as an internal standard. The method was linear in the concentration range of 5–2000 ng/ml for both compounds. The intra-day and inter-day relative standard deviations (RSDs) were less than 15%, and all relative errors (REs) were within 15%. The proposed method enables the unambiguous identification and quantification of these two compounds *in vivo*. This study is the first to determine the 3MBM and 4MBM levels in rat plasma after oral administration of these compounds. These results provide a meaningful basis for evaluating the clinical applications of these medicines.

## Introduction

**Fig 1:**
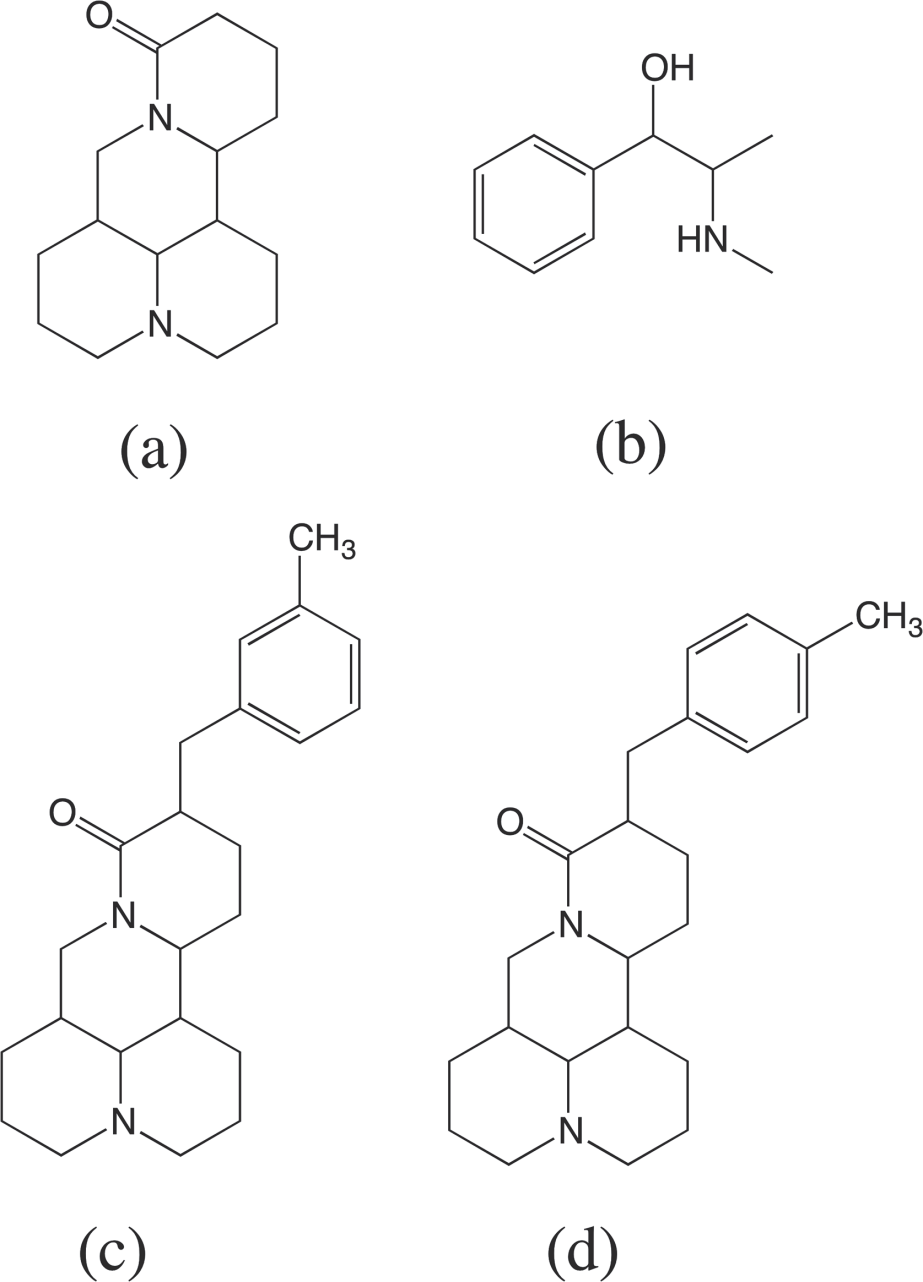
Chemical structures of MT (a), PPD (b), 3MBM (c) and 4MBM (d).

Matrine (MT, Fig. 1A) is a quinolizidine alkaloid isolated from *Sophora alopecuroides*, *Sophora flavescens* or *Sophora subprostrata*, and it has been used extensively in traditional Chinese medicine for the treatment of viral hepatitis, cancer, and cardiac and skin diseases [1–3]. To enhance its pharmacological activities, many MT derivatives have been prepared by previous methods [4], and the biological activity of these derivatives was shown to be significantly different. 14-(3-Methylbenzyl)matrine (3MBM, Fig. 1C) and 14-(4-methylbenzyl)matrine (4MBM, Fig. 1D) are derivatives of MT, and these two MT derivatives share similar physicochemical properties, such as their partition coefficient (logP of approximately 3.93, calculated using Advanced Chemistry Development software). The tumor inhibition rates of 3MBM and 4MBM were 61.83% and 51.15% higher, respectively, than that of MT (39.69%) in a previous pharmacological experiment [5]. To clarify the difference between the activities of 3MBM and 4MBM, a sensitive and accurate analytical method for the determination of 3MBM and 4MBM levels is required to support pharmacokinetic (PK) studies. Recently, much attention has been paid to the absorption and metabolism of MT and oxymatrine, but few studies on the PKs and pharmacodynamics of these compounds using primarily high-performance liquid chromatography-tandem mass spectrometry (HPLC-MS) have been published [6–14]. HPLC-MS ensures high sensitivity for quantification and high specificity in a relatively short analytical time without the need for complete chromatographic resolution of the analytes. However, to the best of our knowledge, previous studies have examined only the administration of MT or oxymatrine, and no study has reported the pharmacokinetics of 3MBM or 4MBM. In the present study, a new HPLC-MS/MS method was developed and validated for the quantification of 3MBM and 4MBM in rat plasma, which is suitable for the investigation of their PK profiles.

## Experimental

### Reagents and chemicals

3MBM and 4MBM (99.8% of purity) were synthesized at the Department of Chemistry and Chemical Engineering of Guangxi University (Nanning, China). Pseudoephedrine hydrochloride (PPD, Fig. 1B, used as an internal standard, IS) with a purity of greater than 99% was obtained from the National Institute for the Control of Pharmaceutical and Biological Products (Beijing, China). Isopropanol was purchased from Caledon (Georgetown, Ontario, Canada). Ethyl acetate was provided by Dikma (Richmond Hill, ON, Canada). LC-grade acetonitrile and methanol were purchased from Fisher Scientific (Pittsburgh, PA, USA). Deionized water was purified using an Alpha-Q water purification system (Millipore, Bedford, MA, USA) and was filtered using 0.20-μm membranes.

### Animals

Male Sprague–Dawley rats weighing 250 to 300 g were purchased from the Laboratory Animal Center of Guangxi Medical University, China. Rats were housed in a pathogen-free facility with a controlled temperature of 24 ± 1°C, a relative humidity of 55 ± 5%, and a 12-h light/dark cycle (7:00 am to 7:00 pm). The animals were acclimated for one week in the Laboratory Animal Center of School of Public Health, Guangxi Medical University before undergoing any experimental procedures. All animals had free access to water and a standard rat diet. Food was withdrawn the night before the experiment; however, water was allowed ad libitum. Free access to food was then resumed 4 h after dosing.

### Ethics Statement

The animals were maintained and the experiments were conducted in accordance with the Institutional Animal Care and Use Committee, Guangxi Medical University, and with the recommendations in the Guide for the Care and Use of Laboratory Animals of the National Institutes of Health. The study was approved by the Animal Care and Use Committee of Guangxi Medical University (Permit Number: SYKG2003–0005). All efforts were made to minimize suffering through the use of post-injury care and monitoring.

### Instruments and conditions

HPLC analysis was performed using the Dionex UltiMate 3000 HPLC equipped with a binary pump, an on-line degasser, an auto-sampler and a column temperature controller. The chromatographic separations were performed using a Hypersil GOLD C18 column (100 mm × 2.1 mm, 1.9 μm particle size) protected by a C18 guard column (10 mm × 2.1 mm, 5 μm) at 40°C. The mobile phase consisted of acetonitrile:0.1% formic acid (65:35, v/v). The flow rate was set at 0.2 ml/min. Aliquots of 2 μl were injected into the HPLC system for analysis.

MS analysis was performed using a Thermo Scientific TSQ Quantum Access MAX triple-stage quadrupole mass spectrometer equipped with an electrospray ionization (ESI) source in the positive-ionization mode. The typical ion source parameters were as follows: spray voltage: 3500 V; sheath gas pressure (N_2_): 20 units; auxiliary gas pressure (N_2_): 5 units; ion transfer tube temperature: 350°C; collision gas (Ar): 1.5 mTorr; Q1/Q3 peak resolution: 0.7 Da; and scan width: 0.002 Da. The samples were analyzed via selective-reaction monitoring (SRM) of the ion pairs at m/z 343 → 148 for 3MBM, m/z 343 → 148 for 4MBM, or m/z 166 → 133 for the IS. The scan dwell time was set at 0.1 s for every channel. All data collected in the centroid mode were acquired and processed using Xcalibur 2.2 software (Thermo Fisher Scientific, Inc., USA).

### Preparation of standards and quality control samples

Stock standard solutions of 3MBM and 4MBM were prepared by dissolving approximately 10 mg of the accurately weighed substance in 100 ml of methanol. Then, these solutions were serially diluted in methanol to generate working standard solutions at the desired concentrations. PPD (10.0 mg) was dissolved and diluted in methanol to yield a stock solution at a concentration of 1.0 mg/ml, which was further diluted in methanol to yield a 5.0 μg/ml IS working solution. All solutions were stored at 4°C and warmed to room temperature before use. Calibration standards were prepared daily by spiking 100 μl of blank plasma with the appropriate working standard solution (50 μl of 3MBM or 4MBM) to obtain concentrations of 5, 10, 50, 100, 500, 1000 or 2000 ng/ml of the corresponding MT derivative. Quality control (QC) samples were prepared at low, intermediate and high concentrations in the same manner.

### Plasma sample preparation

Each plasma sample (100 μl) was mixed with 20 μl of the working IS solution in a 1.5-ml labeled microcentrifuge tube and was vortexed for 5 s. Then, 1000 μl of isopropanol:ethyl acetate (v/v, 25:75) was added, and each sample was vortexed for 1 min. Each tube was subsequently centrifuged at 12,000 × g for 10 min. The organic layer was transferred to another tube and evaporated to dryness at ambient temperature under a gentle stream of nitrogen. The residue was reconstituted in 100 μl of the mobile phase, and 2 μl of each sample was injected into the HPLC–MS/MS system for analysis.

### Method validation

Selectivity was assessed by comparing the chromatograms of six different batches of blank rat plasma to the corresponding spiked rat plasma. Linearity was assessed via weighted (1/x^2^) analysis of the six different calibration curves. Intra- and inter-day precision (the relative standard deviation, RSD) and accuracy (the relative error, RE) were determined by analyzing the low-, intermediate-, and high-concentration QC samples (n = 6) on three different days. The matrix effect was investigated by comparing the peak areas of the analytes in the post-extraction spiked blank plasma samples at low and high analyte concentrations to the corresponding standard solutions. The extraction yield was determined by comparing the mean peak areas of the six extracted samples at low, intermediate, and high QC concentrations to the mean peak areas of the spike-after-extraction samples. Stability was assessed by analyzing the replicates (n = 6) of the low, intermediate and high QC samples during the sample storage and processing procedures. Freeze–thaw stability was determined after three freeze–thaw cycles. Post-preparation stability was estimated by analyzing the QC samples after 24 h at 4°C. Six aliquots of the QC samples were stored at −20°C for 60 days to determine their long-term stability.

### PK study using rats

Aqueous solutions of 3MBM and 4MBM were separately administered to 12 rats via gavage at 25 mg/kg (2.5 mg/ml aqueous solutions), which was equivalent to the dose administered to mice in our previous study [5]. Serial blood samples (0.5 ml) were obtained at 0, 15, 30, 45, 60, 90, 120, 150, 240, 600, 960, and 1440 min after oral administration. All samples were placed in heparinized tubes. After centrifugation at 12,000 rpm and 4°C for 10 min, the plasma was collected and frozen at −20°C until analysis. The PK parameters were estimated according to a non-compartmental model using the TopFit 2.0 software package (Thomae, Germany). The elimination half-life (t_1/2_) was 0.693/k_e_, where k_e_, the elimination rate constant, was calculated by fitting the mean data at four terminal points of the plasma concentration profile to a log-linear regression equation using the least-squares method. The maximum drug plasma concentration (C_max_) and time to C_max_ (T_max_) were directly determined from the observed data. The area under the plasma concentration–time curve from zero to the time of the final measurable sample (AUC_0−t_) was calculated using the linear-trapezoidal rule.

## Results and Discussion

### Chromatography and mass spectrometry

The mobile phase was optimized for sensitivity, speed and peak shapes. Initially, various ratios of methanol to water were used, but poor chromatographs were obtained. Therefore, a solution consisting of acetonitrile:0.1% formic acid in water (v/v, 35:65) was employed. Under the final chromatographic conditions, PPD, one of the candidate compounds used in the previous study [15], displayed retention and ionization similar to 3MBM, and it was utilized as the IS. For sample pretreatment, protein precipitation with methanol or acetonitrile was first attempted due to its simplicity and because examples of its successful use have been reported [7,16]. However, this method resulted in poor extraction efficiency in our study. Thus, we used isopropanol:ethyl acetate (v/v, 5:95) to extract the samples, as previously reported [17], which provided clean extracts and higher yields of both the analyte and the IS. Instead of two extraction cycles, the addition of isopropanol to ethyl acetate (v/v, 25:75) further increased the yield without reducing the purity of the extracts.

**Fig 2:**
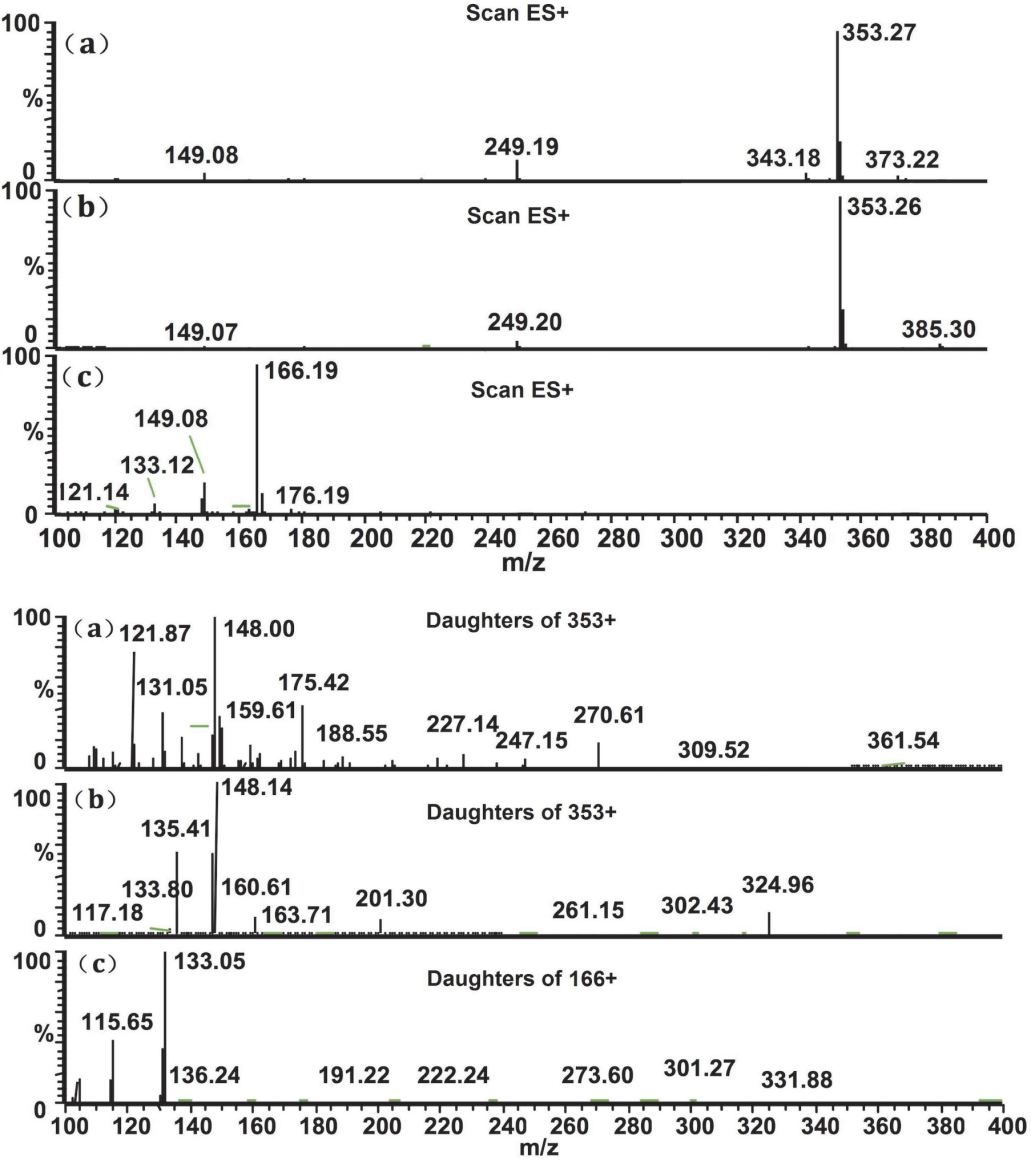
Precursor ion and product ion spectra of 3MBM (a), 4MBM (b) and PPD (c).

The ESI source provided a better response than the APCI source for both analytes. In the precursor ion full-scan spectra, the most abundant ions were protonated molecules [M+H]^+^ at m/z 353, 353 and 166 for 3MBM, 4MBM and the IS, respectively. The MS parameters, such as desolvation temperature, ESI source temperature, capillary and spray voltage, and flow rate of the desolvation gas and auxiliary gas, were optimized to obtain the highest intensities of the protonated molecules of the analytes and the IS. The product ion scan spectra displayed high-abundance fragment ions at m/z 148, 148 and 133 for 3MBM, 4MBM and the IS, respectively (Fig. 2). The collision gas pressure and collision energy of collision-induced decomposition (CID) were optimized to result in maximum responses for the fragmentation of the two analytes. SRM was performed on the precursor → product ion transition at m/z 343 → 148 for 3MBM, m/z 343 → 148 for 4MBM and m/z 166 → 133 for the IS (Fig. 2).

### Method validation

**Fig 3:**
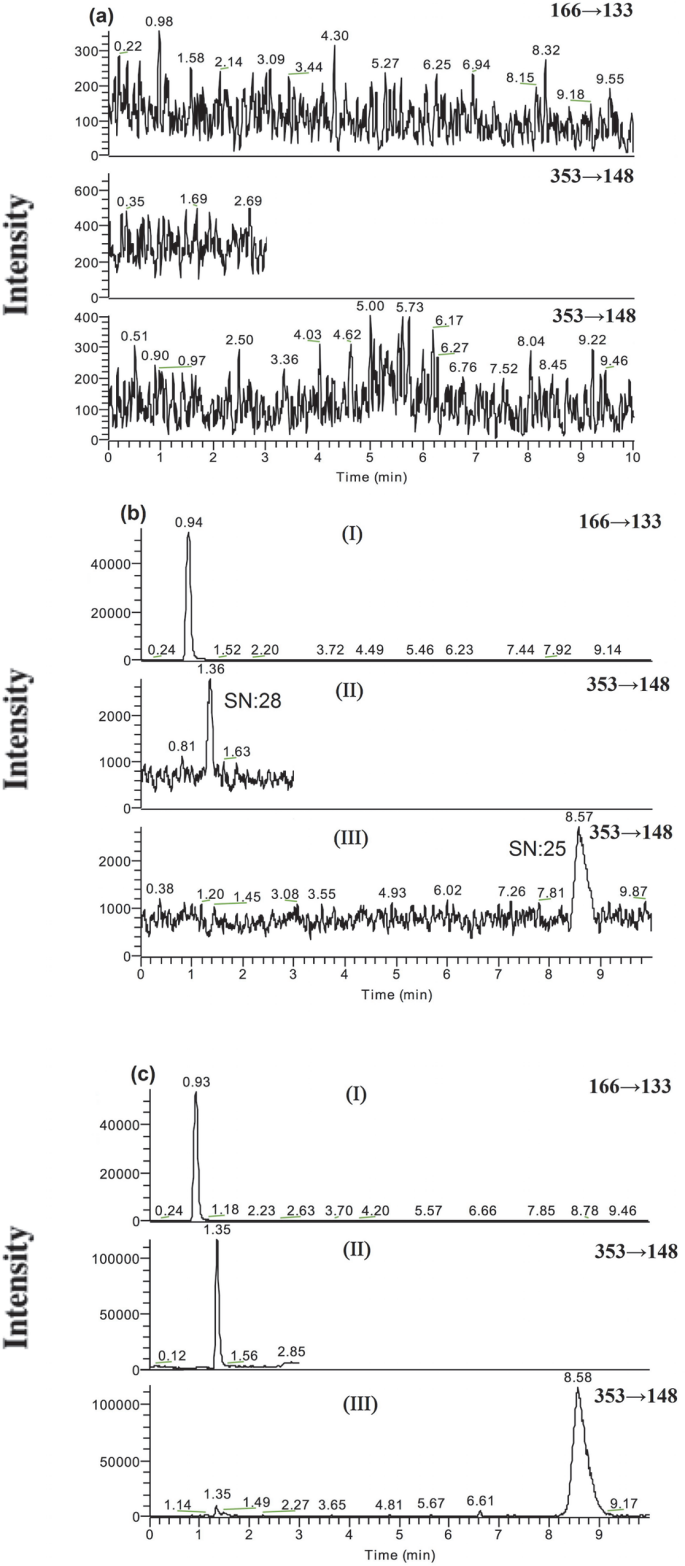
Representative SRM chromatograms of PPD (the IS, I), 3MBM (II) and 4MBM (III) in rat plasma samples: (a) a blank rat plasma sample; (b) a blank rat plasma sample spiked with PPD (1000 ng/ml), 3MBM (5 ng/ml), or 4MBM (5 ng/ml); (C) a rat plasma sample collected 90 min after an oral dose of 3MBM (II) and 4MBM (III) administered to a Sprague–Dawley rat.

**Specificity**. The interference of endogenous plasma constituents with 3MBM, 4MBM and the IS was assessed by inspecting the chromatograms derived from the processed blank plasma samples. Typical chromatograms obtained from blank plasma, blank plasma spiked with a target analyte and the IS, and plasma samples after administration of a target analyte are presented in Fig. 3. The retention times of PPD (IS), 3MBM and 4MBM were approximately 0.92, 1.36 and 8.57 min, respectively. There was no endogenous interference or matrix effect on ionization.

**Linearity and the lower limit of quantification (LLOQ)**. Calibration curves displayed good linearity in the concentration range of 5–2000 ng/ml for 3MBM and 4MBM. The typical calibration curve equations and their correlation coefficients were calculated to be as follows: 3MBM, *y* = 3.95 × 10^−4^ *x* + 7.18 × 10^−3^ (*r* ^*2*^ = 0.9982); 4MBM, *y* = 1.66 × 10^−3^ *x* + 6.14 × 10^−1^ (*r* ^*2*^ = 0.9988). In the regression equation *y* = *ax* + *b*, *x* refers to the concentration of the analyte in serum (ng/ml), and *y* refers to the peak area of the analyte and the IS. A representative chromatogram at the LLOQ is provided in Fig. 3(B). The signal-to-noise ratio (SN) was 28 and 25 for 3MBM and 4MBM, respectively.

**Precision and accuracy**. The intra-day and inter-day precision and accuracy were determined by measuring the QC samples at three concentrations. The intra-day accuracy ranged from −3.66% to −1.19% with an RSD of less than 7.64% for 3MBM and from −1.87% to 1.76% with an RSD of less than 6.37% for 4MBM. The inter-day accuracy ranged from −3.72% to −1.63% with an RSD of less than 9.73% for 3MBM and from −2.20% to 3.16% with an RSD of less than 6.98% for 4MBM. The results indicated that the overall reproducibility of the method was acceptable (Table 1).

**Table 1:** Validation of the intra-day and inter-day assays.

Analyte		Intra-day (*n* = 6)			Inter-day (*n* = 3)		
	Spike concentration (ng/ml)	Concentration (ng/ml)	Accuracy (RE, %)	Precision (RSD, %)	Concentration (ng/ml)	Accuracy (RE, %)	Precision (RSD, %)
3MBM	5 (LLOQ)	5.13	−2.50	7.64	5.19	−3.72	9.73
	800	809.59	−1.19	2.55	826.34	−3.19	4.18
	1600	1660.82	−3.66	4.18	1626.58	−1.63	2.58
4MBM	5	4.93	1.46	6.37	4.85	3.16	6.98
	800	786.15	1.76	3.98	776.78	2.99	5.04
	1600	1630.43	−1.87	4.91	2035.91	−2.20	5.01

**Extraction recovery and ionization**. The mean extraction recoveries determined using six replicates of the QC samples at the low, intermediate and high concentrations were 84.22 ± 5.30% (RSD, 8.4%), 87.51 ± 6.26% (RSD, 8.6%), and 80.40 ± 4.25% (RSD, 7.8%), respectively, for 3MBM; 83.88 ± 5.21% (RSD, 8.1%), 86.23 ± 6.55% (RSD, 7.2%), and 84.85 ± 5.13% (RSD, 9.0%), respectively, for 4MBM; and 94.22 ± 5.46% (RSD, 7.45%) and 93.45 (RSD, 6.23%), respectively, for the IS.

Regarding ionization, the peak area ratios of the two target analytes and the IS after spiking the evaporated plasma samples at three concentration levels were comparable to the neat standard solutions, ranging from 96.7% to 104.5% for 3MBM and from 95.8% to 103.8% for 4MBM, suggesting that the method did not involve any matrix effects.

**Stability**. The stability of 3MBM and 4MBM in the processed samples after freeze–thaw cycles and long-term cold storage (−20°C, 60 days) were evaluated, and the results are summarized in Table 2. The results suggested that the two analytes were stable in the plasma samples for 24 h in an autosampler after preparation, for 60 days in cold storage and for three freeze–thaw cycles; no obvious changes in the concentrations of 3MBM and 4MBM were detected in plasma that was tested within the indicated period under the indicated storage conditions.

**Table 2:** Summary of the stability of MT and TMM in plasma (n = 6, mean ± SD).

Analyte		Stability I^a^		Stability II^b^		Stability III^c^	
	Spike concentration (ng/ml)	Concentration (ng/ml)	R.E. (%)	Concentration (ng/ml)	R.E. (%)	Concentration (ng/ml)	R.E. (%)
3MBM	5	4.94±0.46	-1.02	5.15±0.35	2.87	4.81±0.42	-3.80
	800	791.7±13.6	-1.03	785.4±46.8	-1.82	809.8±30.9	1.22
	1600	1593±39.9	-0.40	1639±98.5	2.46	1660±63.9	3.78
4MBM	5	5.16±0.29	3.20	4.98±0.23	1.32	5.13±0.37	2.60
	800	824.9±37.2	3.11	789.6±67.9	-1.30	787.4±24.1	-1.58
	1600	1577±60.7	-1.42	1583±99.2	-1.04	1636±36.6	2.23

a Refers to post-preparative stability, which was determined in processed samples after storage in an auto-sampler for 24 h.

b Refers to freeze and thaw stability, which was determined in plasma samples after three freeze-thaw cycles.

c refers to long-term cold storage stability, which was determined in plasma samples after 60 days of storage at -20° C.

### PK studies

**Fig 4:**
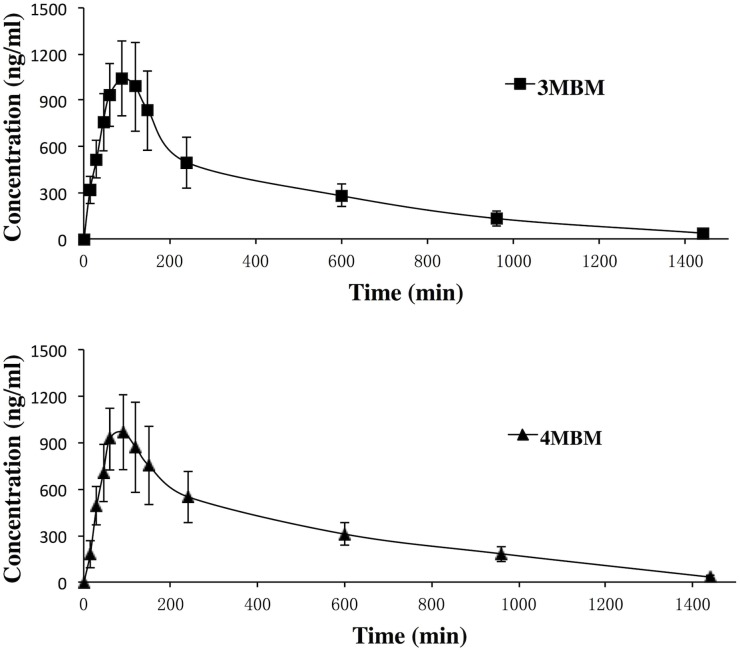
Mean (±SD) plasma concentration–time profiles of 3MBM and 4MBM administered to 12 Sprague–Dawley rats.

The validated analytical method was first employed to study the PK parameters of 3MBM and 4MBM in rats. The mean plasma concentration-time curves following oral administration of 3MBM or 4MBM are presented in Fig. 4.

The PK parameters of 3MBM and 4MBM were calculated according to the non-compartmental method using the TopFit 2.0 software package (Thomae, Germany). The fitted PK parameters are shown in Table 3. After oral administration of 3MBM, its concentration reached peaked at 1053 ± 233 ng/ml and declined rapidly thereafter. After oral administration of the same dose of 4MBM, this analyte was readily absorbed, reaching a C_max_ of 992 ± 275 ng/ml at approximately 95 min (Fig. 4), which was only slightly lower than the value for 3MBM. The C_max_ and T_max_ values of 3MBM and 4MBM following oral administration were not significantly different. Ran [5] evaluated the effect of 3MBM and 4MBM on anti-hepatoma activity in vivo. The results showed that the activity of 3MBM is the same as those of the reference substances of cyclophosphamide, which were higher than that of 4MBM. Hence, it could be speculated that 3MBM is more suitable for activity than 4MBM. Further studies are needed to clarify the mechanisms of 3MBM. In conclusion, this study could provide a reference for modifying the chemical structure of MT.

**Table 3:** PK parameters of 3MBM and 4MBM in rat plasma (n = 6, mean ± SD) after oral administration of 3MBM and 4MBM.

Parameter	3MBM	4MBM
C_max_ (ng/ml)	1053 ± 233	992 ± 275
T_max_ (min)	88 ± 31	95 ± 29
AUC_0-t_ (min ng/ml)	432432 ± 120225	460959 ± 159048
AUC_0-t_ (min ng/ml)	448530 ± 122984	475022 ± 166773
t1/2 (min)	298 ± 35	295 ± 22
K_e_ (1/min)	0.002351 ± 0.000239	0.002365 ± 0.000186
V (ml)	25119 ± 5779	24361 ± 6921
CL (ml/min)	59 ± 13	59 ± 21
MRT_0−t_ (min)	444 ± 15	460 ± 32

## Conclusions

A rapid, sensitive and specific HPLC–MS/MS method was developed and successfully applied for the analysis of 3MBM and 4MBM in rat plasma samples. The precision, extraction yield, matrix effect and stability of this method have been validated. This study is the first to determine the 3MBM and 4MBM levels in rat plasma after oral administration.
